# Too similar, too different: the paradoxical dualism of psychiatric stigma

**DOI:** 10.1192/pb.bp.113.044693

**Published:** 2014-08

**Authors:** Tania Louise Gergel

**Affiliations:** 1 King’s College London

## Abstract

Challenges to psychiatric stigma fall between a rock and a hard place. Decreasing one prejudice may inadvertently increase another. Emphasising similarities between mental illness and ‘ordinary’ experience to escape the fear-related prejudices associated with the imagined ‘otherness’ of persons with mental illness risks conclusions that mental illness indicates moral weakness and the loss of any benefits of a medical model. An emphasis on illness and difference from normal experience risks a response of fear of the alien. Thus, a ‘likeness-based’ and ‘unlikeness-based’ conception of psychiatric stigma can lead to prejudices stemming from paradoxically opposing assumptions about mental illness. This may create a troubling impasse for anti-stigma campaigns.

## The paradox of psychiatric stigma: similar or different, blame or fear

‘If only I could use this to show people that there really was something different about my brain, my life would be so much easier’ - this was the recent reaction of a service user with a history of severe depression to a presentation of magnetic resonance imaging (MRI) images highlighting particularities of the depressed brain. He had experienced discrimination because his condition was viewed not as legitimate illness, but ‘weakness and malingering’, and he expressed a common desire for something to demonstrate a physical difference accompanying mental illness - to display an objective reason for his symptoms, outside of his control. Prejudice against him seems rooted in ideas that he is too weak, lazy, selfish or devoid of willpower to manage the challenges of life like others, despite being, fundamentally, the same. Meanwhile, there is an important theme within anti-stigma work to emphasise the normality, prevalence and universality of mental illness, in the hope that stressing likeness and commonality will lead to a reduction in public fear and alienation.

Stigma is defined as ‘a mark of disgrace’ or as a distinguishing negative token. Thornicroft, for example, calls it ‘a characteristic that individuals possess (or are believed to possess) that conveys a social identity that is devalued, or a mark of disgrace associated with a particular circumstance, quality, or person’ (p. 9ff) and explains how it engenders prejudicial beliefs, which lead to discriminatory behaviour.^1^ The idea of ‘otherness’ is central to psychiatric stigma. Yet my example shows a victim of discrimination actually suggesting that a substantive marker of difference could potentially reverse prejudice. It seems that, for some, psychiatric stigma cannot be eradicated by simply convincing people that mental illness involves no fundamental difference. So we have a perplexing paradox - stigma appears to stem from two opposing beliefs that those with mental illness are both different and also not actually different. Although there may be no simple solution to the problem of psychiatric stigma, a conceptual framework for explaining this central paradox might at least shed some light on the difficulties.

Although clearly multifaceted and complex, psychiatric stigma is also full of puzzling contradictions. It still appears that fear on the one hand and blame on the other dominate discriminatory attitudes towards mental illness. We find ourselves stuck between a rock and a hard place, where decreasing one stigmatising attitude may inadvertently increase another. Biomedical models might reduce blame and promote treatment,^2-4^ but they can increase perceptions of danger, desire for social distance and acceptance of more coercive treatment measures.^5-9^ Stressing affinity or psychosocial factors risks further alienating those with more severe mental illness, minimising the problems they face and taking us full circle back to the idea of mental illness as an inability to manage the stresses and challenges of life, resulting from weakness of personality, moral strength or self-control (not to mention the concomitant dangers of over-pathologising and medicating ‘ordinary’ stress reactions).^10,11^

In this journal, Kingdon argued that even ideas like the one in four mantra do not sufficiently highlight commonality, and recommended replacing ‘mental disorder’ with a more ‘socially inclusive’ stress continuum model of mental illnesses as extreme variants of reaction to stresses faced by everybody.^12^ In response, Braithwaite^13^ maintains that the ‘medicalisation of stress belittles major mental illness’ and that there is no convincing evidence that a number of major mental illnesses are any more likely to be triggered by psychosocial stress than numerous physical conditions. Stigma, he argues, will not be eradicated by simply reconceptualising an illness to remove any biomedical distinguishing factors. Indeed, portraying major mental disorder as part of a normal stress continuum may actually increase stigma by belittling serious medical conditions.^13^ For Kingdon, to defeat stigma, we must emphasise affinity and ‘normality’; for Braithwaite, difference and ‘disorder’. However, a subsequent report suggests that choosing a ‘stressed’ or ‘ill’ model makes very little difference to public perception.^14^ As the authors point out, even a stress model leaves the difficult question of why “‘normal” mentally ill people react differently to stressful events which the majority of people can deal with?’

Even if it seems tempting to downplay difference, this can be a risky strategy. If we suggest that mental illness is not substantively different from ordinary experience, how can we explain and understand its debilitating consequences without recourse to some type of moral judgement?

Moreover, what about symptoms and behaviours to which others cannot relate? Presenting mental illness as purely an extreme variant of ordinary behaviour might engender greater misunderstanding and suspicion surrounding symptoms which cannot be explained accordingly. A blogger from the Time to Change website comments on psychosis: ‘Telling someone you have a mental illness is one thing. Telling them it’s Bipolar is another. Telling them you hear voices, see people who aren’t there and occasionally feel them touching you on the side of your face is quite another thing entirely’.^15^ Another blogger, a woman with schizophrenia, talks about interceding on behalf of a man ‘being almost thrown out of a local coffee shop for looking a bit eccentric when he was unwell’. By comparing mental illness to a physical problem such as a broken limb, she could explain how the owner was being discriminatory and have the unwell man accepted. Without recourse to explanations involving illness and difference, the man would remain ostracised for abnormal, but controllable, behaviour.^16^

Equally, it is often assumed that downplaying difference will help people with mental illness to accept themselves. Metseagharun, writing in support of Kingdon’s argument, says ‘it is of course less bruising to anyone’s ego to accept having a difficulty (or stress) than to accept having a disorder (an implicit indication of socially undesirable or deviant behaviour)’.^17^ But why should this be the case? When someone faces financial ruin after a manic episode or finds themselves unable to work or function, plagued by delusions, anxieties or depression, why would it be reassuring to believe that the cause of these devastating problems was essentially their own inability in coping with stress? Conversely, a medical understanding may often help those with mental illness reach acceptance, find treatment and gain more control: ‘accepting and gaining insight of my illness has enriched my life’, comments a Time to Change blogger;^18^ another blogger understands his condition as similar to his hay fever: conditions which require medication and ‘won’t go away of their own accord’.^18-20^

## Towards a likeness-based and unlikeness-based conception of stigma

The stigma debate must contend with such puzzling difficulties.^21^ Although increasing awareness and understanding are generally agreed to be crucial to battling stigma and discrimination, current initiatives still struggle (pp. 243-245).^1^ For example, the UK Time to Change campaign, despite its achievements, has failed so far to fulfil significantly its key aim to improve knowledge or behaviour among the general public.^22^

If we are want to promulgate knowledge or institute policy changes to reverse structural discrimination, without engendering negative assumptions, perhaps we need a clearer understanding of such misunderstandings and contradictions. It is here that a conceptual framework, which explicates psychiatric stigma as not just multidimensional or complex, but inherently paradoxical, might help. As previously mentioned, stigma stems from notions of otherness. With psychiatric stigma, however, the two major kinds of difference believed to differentiate the mentally ‘ill’ and ‘healthy’ seem to be in direct opposition, their being based on converse notions of unlikeness and likeness. Many people with mental illness will have experienced both types of prejudice, even from the same sources, despite apparent self-contradiction.

Unlikeness-based stigma is probably the more easily understood, with mental illness seen as making people intrinsically different, somehow ‘alien’ and thus easily feared, ridiculed or restrained. Yet, although we may fear those whom we see as different, those who cannot be understood, predicted or controlled, it would seem illogical to apportion blame for actions or behaviour unless we believe the agent to share common ground and equal capacities as ourselves for acting differently.

The root of attitudes such as blame may lie in stigma based on another equally disturbing view, which we might term likeness-based stigma and which stems from the idea of similarity and a view of mental illness as infirmity of character rather than legitimate illness. Whereas unlikeness-based prejudice suggests that mental illness is a defect in the very qualities which define a normal human being, likeness-based stigma implies a problem that is moral rather than substantive or biological - that those with mental illness share the same biological and environmental factors as others, but lack the strength of character to deal with them. Because, however one looks at it, mental illness, like any health condition, sets aside those who experience it from those who do not. In the absence of other explanations, on the likeness-based model, people may well construe the differences in behaviours and experiences as stemming from some type of moral inferiority.

## Examples of likeness-based stigma

Much anti-stigma work focuses on unlikeness-based stigma, even though likeness-based stigma can be seen to be extremely prevalent and significant.

Although perceptions of mental illness vary in different societies and cultures, the view that the individual is in some way to blame for their condition is very common. In *Shunned*, Thornicroft has suggested that a perceived lack of willpower and notion of blame may even be the one thing which comes close to being a unifying global feature (pp. 176-179).^1^ He lists some common myths particular to mental illness and derived from real experiences of discrimination (p. 187).^1^ Although the idea of fundamental difference is very much at the heart of views such as ‘all schizophrenics are dangerous and violent’, many other beliefs point towards a ‘likeness-based’ view that people with mental illness are fundamentally the same, but lazy, weak or incapable, and therefore responsible and blameworthy for their condition: ‘depression results from personality weakness or character flaw, and people could snap out if they tried harder’; the mentally ill are ‘lazy and not trying’; ‘mental illness is brought on by weakness of character’; ‘if you have a mental illness, you can will it away, and being treated for a psychiatric disorder means you have in some way failed or are weak’; ‘psychiatric disorders are not true medical illnesses like diabetes’.^1^

Nor are such ideas restricted to private contexts. Discrimination by mental health professionals remains widespread^23^ and service users often find themselves being held responsible for their conditions. An example is a common experience for service users, known as diagnostic overshadowing, which could be understood to stem from similar attitudes. This occurs when ‘physical illness signs and symptoms are misattributed to psychiatric disorder’, so that physical symptoms appear to be judged as either psychological in origin or of exaggerated severity, and which may result in a lack of appropriate medical attention.^24-26^ Reflected here is an underlying attitude that psychiatric patients faced with ordinary challenges lack the moral strength to tolerate them as well as others do and are therefore in some way blameworthy or responsible for their symptoms and behaviours.

A couple of examples reveal that similar ideas can also be found within academic medicine. A 2009 *British Journal of Psychiatry* editorial on the moral content of psychiatric treatment argues that ‘psychiatric treatment can enhance human morality’ and that ‘helping patients to be more virtuous is a proper concern of psychiatry’.^27^ The same authors subsequently argue elsewhere that for conditions such as depression, willpower to change behaviour has a crucial and direct effect on the condition itself - ‘patients must decide to behave differently and have the will to do it’ - whereas medical interventions, even in the case of depression, are presented as secondary means to ‘bolster resolve and willpower’.^28^ They sharply differentiate these from conditions such as diabetes or cancer, where willpower only affects factors such as adherence to treatment.^28^ In a very different context, Schlaepfer *et al*,^29^ in response to concerns that deep brain stimulation might affect personality, state that, for psychiatric conditions, ‘modification of mood and cognitive behaviour - and thus important elements of personality - is not an unwanted, coincidental side effect, but rather the main intended outcome.’^29^ Although it is hard to imagine a psychiatric intervention underpinned by a more neurological model than psychiatric neurosurgery, the authors still seem to conflate alterations in mood and cognitive behaviour caused by treatment-resistant major depression or obsessive-compulsive disorder with the service user’s personality and see treatment in terms of modification of personality. Without intending to suggest prejudice in either set of authors or that this brief discussion reflects the full complexity of the authors’ ideas, both pieces lend themselves to the idea that mental illness is, to a significant degree, constituted by defects of personality or morality in individuals, and that the aim of psychiatric treatment is to rectify these weaknesses, rather than to treat an illness viewed as independent from the true personality of the affected individual.

## What are the implications?

Highlighting the importance of likeness-based stigma does not mean that an unlikeness-based view of mental illness as abnormal, alien and to be feared is not a prevalent and major cause of discrimination. Such attitudes are all too clear in recent examples such as the ‘mental patient’ and ‘psycho ward’ outfits withdrawn from two major supermarket chains^30^ and the controversy over the sensationalising and inaccurate article in a major tabloid on numbers of people killed by ‘mental patients’.^31^

However, we should not also undervalue apparent benefits of illness models which entail ‘difference’ or assume that the solution is simply to hide or de-emphasise uncomfortable aspects of mental disorder.^32^ For example, illness models seem to have generated an increase in willingness to seek treatment, whereas major progress against structural discrimination, such as the decriminalisation of suicide, appears largely to have resulted from official recognition of suicide as stemming predominantly from mental illness. Moreover, stress continuum, prevalence or recovery models could lead to decreased public spending on health services, through viewing the service user as primarily responsible for their own recovery.^33^

The formulation of psychiatric stigma as presented here suggests that it emerges from two perspectives on those with mental illness that appear to be paradoxically opposed. This would seem to pose an immense difficulty for anti-stigma campaigns. Highly sophisticated messages and interventions have been developed in this field, but I suggest that there might be value in thinking through the implications of the paradox. Key questions would then be: if one accepts that there are important differences between the experiences and behaviours of people with a mental illness and the rest of the population, how can the negative evaluations of those differences be challenged? And, given that these negative evaluations may well remain negative, for most of the differences would not be deemed desirable if given the choice, how can the link between these negative evaluations and stigma be broken?

## References

[1] Thornicroft G *Shunned: Discrimination against People with Mental Illness*. Oxford University Press, 2006

[2] Kendell RE The distinction between mental and physical illness. *Br J Psychiatry* 2001; 178: 490–3 11388962 10.1192/bjp.178.6.490

[3] White PD Rickards H Zeman AZJ Time to end the distinction between mental and neurological illnesses. *BMJ* 2012; 344: e3454 22628005 10.1136/bmj.e3454

[4] Craddock N Antebi D Attenburrow M-J Bailey A Carson A Cowen P Wake-up call for British psychiatry. *Br J Psychiatry* 2008; 193: 6–9 18700211 10.1192/bjp.bp.108.053561

[5] Pescosolido BA Martin JK Long JS Medina TR Phelan JC Link BG ‘A disease like any other’? A decade of change in public reactions to schizophrenia, depression, and alcohol dependence. *Am J Psychiatry* 2010; 167: 1321–30 20843872 10.1176/appi.ajp.2010.09121743 PMC4429867

[6] Corrigan PW Watson AC At issue: stop the stigma, call mental illness a brain disease. *Schizophr Bull* 2004; 30: 477–9 15631240 10.1093/oxfordjournals.schbul.a007095

[7] Schomerus G Schwahn C Holzinger A Corrigan PW Grabe HJ Carta MG Evolution of public attitudes about mental illness: a systematic review and meta-analysis. *Acta Psychiatr Scand* 2012; 125: 440–52 22242976 10.1111/j.1600-0447.2012.01826.x

[8] Read J Haslam N Sayce L Davies E Prejudice and schizophrenia: a review of the ‘mental illness is an illness like any other’ approach. *Acta Psychiatr Scand* 2006; 114: 303–18 17022790 10.1111/j.1600-0447.2006.00824.x

[9] Angermeyer MC Holzinger A Carta MG Schomerus G Biogenetic explanations and public acceptance of mental illness: systematic review of population studies. *Br J Psychiatry* 2011; 199: 367–72 22045945 10.1192/bjp.bp.110.085563

[10] Maj M From ‘madness’ to ‘mental health problems’: reflections on the evolving target of psychiatry. *World Psychiatry* 2012; 11: 137–8 23024663 10.1002/j.2051-5545.2012.tb00113.x PMC3449349

[11] Nassir Ghaemi S Why antidepressants are not antidepressants: STEP-BD, STAR^*^D, and the return of neurotic depression. *Bipolar Disord* 2008; 10: 957–68 19594510 10.1111/j.1399-5618.2008.00639.x

[12] Kingdon D Everybody gets stressed . . . it’s just the way we react that differs. *Psychiatr Bull* 2009; 33: 441–2

[13] Braithwaite R Medicalisation of stress belittles major mental illness (letter). *Psychiatrist* 2010; 34: 115

[14] Luty J Easow JM Mendes V Stigmatised attitudes towards the ‘stressed’ or ‘ill’ models of mental illness. *Psychiatrist* 2011; 35: 370–3

[15] Time to Change. My experience of psychosis and bipolar: ‘coming out’ to friends, family and employers (blog). Available at http://www.time-to-change.org.uk/blog/my-experience-psychosis-bipolar (accessed 3 Mar 2014).

[16] Time to Change. There aren’t enough positive stories about people living with schizophrenia (blog). Available at http://www.time-to-change.org.uk/blog/not-enough-positive-stories-about-people-living-with-schizophrenia (accessed 3 Mar 2014).

[17] Metseagharun T We all have thought processing difficulties from time to time . . . it’s just the way we react that differs (letter). *Psychiatrist* 2010; 34: 73

[18] Time to Change. Some believe that men should maintain a stiff upper lip (blog). Available at http://www.time-to-change.org.uk/blog/some-believe-men-should-maintain-stiff-upper-lip (accessed 3 Mar 2014).

[19] Time to Change. Nobody is ashamed to admit that they have hayfever (blog). Available at http://www.time-to-change.org.uk/blog/nobody-is-ashamed-to-admit-that-they-have-hayfever (accessed 3 Mar 2014).

[20] Noakes S Living with depression: the reality. In *Every Family in the Land: Understanding Prejudice and Discrimination against People with Mental Illness* (ed. Crisp AH): 54–7 Royal Society of Medicine Press, 2004

[21] Sayce L *From Psychiatric Patient to Citizen: Overcoming Discrimination and Social Exclusion*: p. 280 Macmillan, 2000

[22] Smith M Anti-stigma campaigns: time to change. *Br J Psychiatry* 2013; 202 (suppl 55): s49–50 10.1192/bjp.bp.113.126813 23553694

[23] Corker E Hamilton S Henderson C Weeks C Pinfold V Rose D Experiences of discrimination among people using mental health services in England 2008-2011. *Br J Psychiatry* 2013; 202 (suppl 55): s58–63 10.1192/bjp.bp.112.112912 23553696

[24] Jones S Howard L Thornicroft G ‘Diagnostic overshadowing’: worse physical health care for people with mental illness. *Acta Psychiatr Scand* 2008; 118: 169–71 18699951 10.1111/j.1600-0447.2008.01211.x

[25] Thornicroft G Rose D Kassam A Discrimination in health care against people with mental illness. *Int Rev Psychiatry* 2007; 19: 113–22 17464789 10.1080/09540260701278937

[26] Van Nieuwenhuizen A Henderson C Kassam A Graham T Murray J Howard LM Emergency department staff views and experiences on diagnostic overshadowing related to people with mental illness. *Epidemiol Psychiatr Sci* 2013; 22: 255–62 23089191 10.1017/S2045796012000571 PMC8367326

[27] Pearce S Pickard H The moral content of psychiatric treatment. *Br J Psychiatry* 2009; 195: 281–2 19794191 10.1192/bjp.bp.108.062729

[28] Pearce S Pickard H Finding the will to recover: philosophical perspectives on agency and the sick role. *J Med Ethics* 2010; 7 31: jme.2010.035865 10.1136/jme.2010.035865 20675734

[29] Schlaepfer T Lisanby S Pallanti S Separating hope from hype: some ethical implications of the development of deep brain stimulation in psychiatric research and treatment. *CNS Spectr* 2010; 15: 285–7 20448518 10.1017/s1092852900027504

[30] BBC News UK. Asda and Tesco withdraw Halloween patient outfits. 26 9 2013 Available at www.bbc.co.uk/news/uk-24278768 (accessed 3 Mar 2014).

[31] Chalabi M The Sun says 1,200 people have been killed by ‘mental patients’ - is it true? (blog). *Guardian* 2013; 7 Oct. Available at www.theguardian.com/society/reality-check/2013/oct/07/sun-people-killed-mental-health-true (accessed 3 Mar 2014).

[32] Phelan JC Genetic bases of mental illness - a cure for stigma? *Trends Neurosci* 2002; 25: 430–1 12127761 10.1016/s0166-2236(02)02209-9

[33] Angermeyer M Schomerus G A stigma perspective on recovery. *World Psychiatry* 2012; 11: 163–4 23024670 10.1002/j.2051-5545.2012.tb00120.x PMC3449353

